# Efficacy and Safety of Zero-Fluoroscopy Approach during Catheter Ablation of Accessory Pathway

**DOI:** 10.3390/jcm11071814

**Published:** 2022-03-25

**Authors:** Dariusz Rodkiewicz, Edward Koźluk, Agnieszka Piątkowska, Aleksandra Gąsecka, Krzysztof Krzemiński, Grzegorz Opolski

**Affiliations:** 1 Department of Cardiology, Medical University of Warsaw, 02-091 Warsaw, Poland; ekozluk@vp.pl (E.K.); agnes.piatkowska@wp.pl (A.P.); aleksandra.gasecka@wum.edu.pl (A.G.); grzegorz.opolski@wum.edu.pl (G.O.); 2 Chair of Emergency Medicine, Wroclaw Medical University, 02-091 Wrocław, Poland; 3 Department of Applied Physiology, Mossakowski Medical Research Centre, Polish Academy of Sciences, 02-106 Warsaw, Poland; k.krzeminski@uthrad.pl

**Keywords:** catheter ablation, fluoroscopy elimination, electroanatomic mapping, accessory pathway

## Abstract

Background: Catheter ablation (CA) is a safe and efficient treatment in patients with an atrioventricular accessory pathway (AP). Electroanatomical mapping (EAM) systems are useful during CA of AP, especially for reducing fluoroscopy. There are limited data about the feasibility of CA procedures performed with the use of the EAM system entirely without fluoroscopy in adults with AP. The aim of the study is to assess the feasibility, efficacy and safety of CA with the use of EAM without fluoroscopy, compared to CA with EAM and fluoroscopy in patients with AP. Methods: The study included 83 consecutive patients (age 38.25 ± 15.8 years), who were subjected to CA for AP. In 40 patients CA was performed with the use of EAM without fluoroscopy (EAM group), and in 43 patients CA was performed with EAM and fluoroscopy (control group). Baseline characteristics, procedure parameters and complications were obtained from the medical records. Data on permanent success rate was obtained after the mean follow-up time of 1 year. Primary outcomes were acute procedural success rate, long term success rate at 1-year follow-up and complications. Secondary outcomes were the procedure time and number of applications. Results: There were no statistically significant differences in baseline characteristics between the groups, except for the AP locations. Right-sided AP was more common in the EAM group, while left-sided AP was more common in the control group (*p* = 0.007 and *p* = 0.004, respectively). Acute procedural success was achieved in 38 patients (95.0%) in the EAM group and in 39 patients (90.7%) in the control group (*p* = 0.449). Long term success rate was achieved in 36 patients (90.0%) in the EAM group and in 36 (83.7%) patients in the control group (*p* = 0.399). There was one minor complication in the form of RBBB in the EAM group (*p* = 0.138). The mean procedure time was shorter in the EAM group compared to the control group (93.0 ± 58.3 min vs. 127.6 ± 57.5 min; *p* = 0.009). Conclusions: CA of both right-sided and left-sided AP completely guided by EAM without the use of fluoroscopy is feasible, safe and effective.

## 1. Introduction

Catheter ablation (CA) has become the established therapy for patients with symptomatic accessory pathway (AP) with an overall success rate ranging from 67 to 100% [1]. Fluoroscopy is a standard navigation guide used to perform both radiofrequency (RF) catheter ablation and cryoablation [2]. The limitations of fluoroscopy navigation include limited projections and lack of possibility to sign the position of the catheters or to create electroanatomic maps. Besides, fluoroscopy navigation is associated with exposure to ionising radiation [2]. In order to facilitate the ablation procedures and minimise the radiation exposure, an electroanatomic mapping (EAM) system was introduced [3]. EAM allows for the creation of the maps of the heart chambers, precise catheter navigation and reduced radiation exposure [4]. EAM proves useful as an adjunct to conventional fluoroscopy in patients with complex arrhythmias such as atrial fibrillation, atypical atrial flutter and ventricular tachycardia [5,6,7], and it is the only navigation system in the paediatric population with AP [4,8,9,10,11]. However, there are limited data about the safety and efficacy of the CA procedures performed with use of the EAM system entirely without fluoroscopy in adults with AP.

The main aim of this study is to assess the feasibility, efficacy and safety of CA with the use of EAM without fluoroscopy, compared to CA with the use of EAM and fluoroscopy in patients with AP.

## 2. Materials and Methods

### 2.1. Study Design and Study Population

Among 83 consecutive patients who underwent CA for AP, we retrospectively selected a group of 40 patients (mean age 35.5 ± 15.9 years) who had CA with the use of EAM, without fluoroscopy (EAM group), and a group of 43 patients (mean age 41 ± 15.8 years) who have undergone CA under EAM and fluoroscopy navigation (control group).

CA in both groups was performed by the same team. Demographic and clinical data, arrhythmia characteristics, procedural characteristics (procedure duration, ablation duration, number of applications, fluoroscopy duration and dose area product), procedural success rates and complications were taken from the medical records. Procedure duration was measured from start of local anaesthesia to removal of venous sheaths. Ablation duration was defined as the summary time of applications. Fluoroscopy time was defined as the total duration of fluoroscopy during the procedure. Acute procedural success was defined as no evidence of AP conduction at the end of the procedure. Major complications included: death, cardiac tamponade, permanent atrioventricular (AV) block, major bleeding, thromboembolic event, myocardial infarction and aorta dissection. Minor complication included vascular access complications (groin hematoma, pseudoaneurysm, arteriovenous fistula), bundle branch block (right—RBBB or left—LBBB), prolongation of PR distance for more than 200 milliseconds and temporary AV block. Acute success rate was assessed 30 min after the last application. The long-term procedural success rate was evaluated at the follow-up time of 1 year.

### 2.2. Electrophysiological Study

All patient signed written informed consent for electrophysiological examination (EPS) and CA before the procedure. Antiarrhythmic drugs were discontinued for a period of at least 5 half-lives prior to procedure. Almost all patients (95%) with AP underwent an EPS to obtain baseline electrophysiological characteristics including incremental atrial pacing (IAP), programmed atrial pacing, incremental ventricular pacing (IVP), programmed ventricular pacing and burst with cycle length (CL) 330 ms, 300 ms and 273 ms from atrium and ventricle. Therefore, steerable decapolar catheter (Hagmed, Rawa Mazowiecka, Poland) was introduced to coronary sinus and to right ventricle quadripolar non-steerable catheter (Hagmed, Rawa Mazowiecka, Poland). In all patients, anterograde and retrograde effective refractory period through the AP and AV node was assessed during EPS. For EPS, vascular access from the right femoral vein was used.

For right-sided AP, the right femoral vein was the only access. For left-sided AP, retrograde approach was used in all patients in the EAM group. In the fluoroscopy group, three different approaches were used: a transseptal approach through patent foramen ovale, a retrograde aortal access after the right femoral artery puncture and an access from the CS, at the discretion of the operator. 

We used both irrigated and non-irrigated ablation catheters. The choice of the catheter depended on the type of AP. For the right-sided parahisian AP, we used non-irrigated ablation catheters or cryoablation to decrease the risk of atrioventricular block. For the left-sided AP, we mostly used the irrigated ablation catheters to decrease the likelihood of thrombus formation and create larger lesions. 

Intravenous unfractionated heparin in a bolus dose of 100 U/kg at an infusion rate of 1000 U/h was administered to all patients requiring CA in the left side of the heart. In addition, the activated clotting time (ACT) was monitored, during the left-sided AP ablation, with the target ACT above 300 s.

### 2.3. Fluoroscopy Navigation

Conventional fluoroscopic mapping was performed with X-ray system (GE Healthcare Worldwide) or angiography system (Siemens Artis Zee, Erlangen, Germany) utilizing the customized settings for each patient to achieve minimal radiation dose compatible with appropriate image quality. Fluoroscopy was used to allow visualization of catheter placement and stabilization during ablation. Up to three catheters were used: quadripolar or decapolar catheter (Hagmed, Rawa Mazwiecka, Poland) located in the right atrium (RA), coronary sinus (CS) and right ventricle (RV). The ablation catheter was inserted after the EPS. During CA with fluoroscopy (control group), medical staff wore lead aprons. 

### 2.4. Electroanatomic Mapping

The EAM was performed with the use of two systems: CARTO (Biosense Webster, Diamond Bar, CA, USA) and EnSite NavX mapping system (Abbot, St. Paul, ML, USA), depending on their availability in the department. CARTO is a magnetic-based three-dimensional electroanatomic mapping system. The system requires the use of proprietary catheters containing magnetic sensors to localize the catheter position within the electromagnetic field generated under the patient’s thorax with the accuracy of 0.54 ± 0.05 mm. In the present study, we used both anatomical and activation maps. EnSite NavX system is an impedance-based technology which detects the voltage gradient between the three pairs of surface electrodes located on the patient’s thorax and displays the catheter position relative to a reference intracardiac electrode, along with creating anatomical, activation and voltage map [12,13]. EnSite NavX allows the catheters to be followed from their femoral venous or arterial sheaths. The system does not require proprietary catheters and allows visualization of many catheters including cryoablation or RF catheters. The resolution of 0.7 ± 1.5 mm in the chamber geometry allows for precise mapping [14].

### 2.5. Catheter Ablation

RF catheter ablation (RFCA) was conducted with the fluoroscopic or EAM system. RF energy was delivered in the temperature control mode, using an EP-Shuttle generator (Stockert, Bar Diamond, CA, USA) or RF generator (Atakr, Medtrocic, Minneapolis, MN, USA) with a power output of 20–60 W and a temperature of 60 °C. RF ablation was performed using non-flow catheters of 6 to 8 French tip catheters. Cryoablation was performed with 7 French Freezor (Medtronic) cryoablation catheter following cryomapping procedure at −30 °C to control the efficacy of the application and to avoid electrophysiological complications. The temperatures during cryoablation reached less than −70 °C. Ablation procedure was considered successful if pre-excitation was observed and no supraventricular tachycardia could be induced for >30 min after the last ablation pulse, neither under basal conditions nor with intravenous isoprenaline. Additionally, the atrioventricular block was documented following intravenous adenosine administration. All patients underwent a post-procedural echocardiogram to exclude pericardial effusion or other acute complications. Other minor and major complications that occurred up to the hospital discharge were recorded.

### 2.6. Follow-Up

All patients had a follow-up visit 4–12 weeks after CA to obtain the updated medical history and document the absence of the index arrhythmia in 24-h Holter electrocardiogram. Additionally, they were contacted by phone after 1 year of follow-up to collect data on long-term procedural success.

### 2.7. Statistical Analysis

Statistical analysis was performed using Statistical Package for the Social Sciences (SPSS—Statistics for Windows, Version 23.0; Armonk, NY, USA, IBM Corp). The data were presented as means of frequency and percentage with standard deviations (SD). Continuous variables were tested for central tendency using Kolmogorov–Smirnov test. Normally distributed continuous variables are expressed as means +/− standard deviation (SD). Non-normally distributed data are expressed using the median value and range. The Chi2 test was used to compare categorical variables. The Student’s t-test or Mann–Whitney U test were used for continuous variables with and without normal distribution, respectively. A two-tailed *p*-value of <0.05 was considered statistically significant.

## 3. Results

### 3.1. Patients’ Characteristics

The characteristics of patients are presented in Table 1. There were no statistically significant differences in baseline demographic and clinical characteristics between the groups, except for the AP locations. Right-sided AP were more common in the EAM group, while left-sided AP more common in the control group (*p* = 0.007, *p* = 0.004, respectively). In the EAM group, there were two cases of left lateral AP, one case of left anterolateral AP and one case of left posterolateral left sided AP. In the control group, there were all types of left sided AP. In the EAM group, there were three pregnant women.

### 3.2. Procedural Data 

Procedural data and results are presented in Table 2. The frequency of isolated RF ablation, cryoblation and combined RF with cryoblation were comparable between the groups, with RF ablation being the most frequent in the EAM group (31/40; 77.5%), when compared with the control group (36/43; 83.7%), *p* = 0.473). There were 25 (62.5%) first-time CA procedures in the EAM group and 27 (62.8%) in the control group (*p* = 0.978). The mean procedure duration was substantially shorter in the EAM group (93.0 ± 58.3 min), compared to the control group (127.6 ± 57.5 min; *p* = 0.009). The mean energy application time and number of energy applications were similar in both groups (*p* = 0.884, *p* = 0.174, respectively). Acute procedural success was achieved in 38 (95.0%) patients in the EAM group and in 39 (90.7%) patients in the control group (*p* = 0.449). Long-term success rate was achieved in 36 (90.0%) patients in the EAM group and in 36 (83.7%) patients in the control group (*p* = 0.399). There were no major complications. There was one minor complication in the form of RBBB in the EAM group (*p* = 0.138). 

Regarding the results of the left-sided procedures, 4/4 procedures were successful in the EAM group (100%) and 14/16 were successful in the control group (88%). There was one minor complication (RBBB) in the EAM group.

## 4. Discussion

The main finding of the present study is that CA of both right-sided and left-sided AP completely guided by EAM without the use of fluoroscopy is feasible, safe and effective. 

The use of fluoroscopy could be entirely excluded in 40 out of 83 (48%) of our AP patients, although sometimes in the event of anatomical difficulties the operator was forced to use fluoroscopy. Procedures without fluoroscopy were shorter than those with fluoroscopy, which may be due to the less complex AP anatomy in patients who underwent CA without fluoroscopy, as indicated by a larger number of patients with right-sided AP in this group compared to the control group.

The results of our study are consistent with the previous studies, confirming the feasibility, efficacy and safety of a completely non-fluoroscopic approach of CA procedures for right-sided and left-sided AP in adults. Earley et al., (2006) compared the use of Ensite NavX and CARTO with that of conventional fluoroscopically guided catheter localization in patients referred for catheter ablation of a wide variety of arrhythmias and found that the Ensite NavX and CARTO procedures reduce X-ray exposure without compromising the duration, effectiveness or safety of the procedure. However, it should be noted that there was no subanalysis on the efficacy and safety of the non-fluoroscopic approach in the subgroup undergoing ablation for AP [8]. Similarly, studies in young and middle-aged patients showed the feasibility, safety, and efficacy of non-fluoroscopic RFCA of supraventricular tachyarrhythmias using the EnSite NavX system. However, among 47 patients undergoing non-fluoroscopic RFCA guided by the EnSite NavX system, there were only 16 patients with AP [6]. Likewise, a 6-year prospective study of 328 patients referred for RFCA of regular supraventricular tachycardia (SVT) confirmed earlier reports that RFCA with an approach completely guided by the Ensite-NavX electroanatomical navigation system without the use of fluoroscopy is feasible, safe and effective [12]. Most of the non-fluoroscopic CA procedures in the hitherto studies focused on RF ablation with the use of only selected EAM systems (CARTO or EnSite NavX), usually in AVNRT patients [13]. Contrary to those studies, the present study demonstrated the feasibility of the zero-fluoroscopy CA approach in the well-defined population with AP. In the present study, CARTO (70%) and EnSite NavX (30%) systems were used both in the EAM group and the control group. Interestingly, eight (20%) procedures in the EAM group and six (14%) procedures in the control group were successfully performed using cryoablation, which proves the safety of EAM without fluoroscopy, regardless of the system and type of ablation used. This is in line with the findings of other authors who have also shown that cryoablation without fluoroscopy is effective and safe for AP located in critical septal areas (near the AV node or bundle of His) [14,15]. EAM eliminates radiation exposure during ablation of less complex supraventricular arrhythmias, such as those mediated by AP, while providing procedural efficacy and safety. Thus, it extends the indications for CA in patients who usually avoided it, such as pregnant women, patients with a history of cancer or haematological diseases or patients with immune system defects [16,17,18]. According to BEIR Report VII (Committee Biological Effects of Ionising Radiation) and IRCP Publication 103 (International Commission on Radiological Protection), the best model to estimate radiation risk is the linear-no-threshold (LNT) model [19,20]. This model supports the concept that no dose of radiation, even the smallest, can be considered completely safe. Based on statistical models, it has been shown that the risk of mortality from cancer caused by typical cardiac intervention is lower than 1%. [19]. Nevertheless, the necessity to perform repeated procedures in a certain number of cases may result in a significant cumulative effective doses and increased risk of cancer. Additionally, it seems likely that the linear-non-threshold model underestimated the risk of radiation due to the phenomenon of low-dose hypersensitivity [21]. Based on this rationale, the use of non-standard fluoroscopy settings or the implementation of a protocol at the lowest possible level (ALARA) for electrophysiological procedures seem to be insufficient to provide complete protection for both the patient and laboratory staff. It should be noted, however, that several studies have questioned the reliability of LNT and postulated the existence of practical thresholds for carcinogenesis [22,23,24,25]. This requires further research to determine the physical nature of the low-dose radiation.

## 5. Limitations

This was a retrospective cohort study with a relatively small number of patients. Consequently, the data were sometimes limited by the completeness of the medical records. In addition, the non-randomized study design might be associated with potential selection bias of patient allocation to EAM or standard methods. The results of this study cannot be indirectly extrapolated to other electrophysiological laboratories because they come from a single centre. The heterogeneity of the study population, including patients with right-sided and left-sided AP undergoing initial and repeat procedures with the two EAM systems and different ablation methods, may have influenced the results of our study. 

## 6. Conclusions

A three-dimensional EAM system enables a safe and effective zero-fluoroscopy AP ablation in all locations. The procedures without the use of fluoroscopy were shorter than those with fluoroscopy. Given that there is no safe radiation dose, the elimination of fluoroscopy is desirable not only for complex ablation procedures but also for simpler arrhythmic substrates such as AP. Although our results indicate that both right- and left-sided AP can be treated without the use of fluoroscopy, the conclusions regarding the left-sided AP should be interpreted with caution and further investigated due to the small number of patients in the left-sided subgroup. The promising results of our study inspire further research on the usefulness of EAM in various patient populations.

## Figures and Tables

**Table 1:** Characteristics of patients (the values are means ± standard deviation). AP—accessory pathway, EAM—electroanatomic system.

Baseline Characteristics	EAM Group, *n* = 40	Control Group, *n* = 43	*p*
Gender (male)	21.0 (53.4%)	27.0 (62.8%)	0.343
Age (years)	35.5 ± 15.9	41.0 ± 15.8	0.154
Hypertension	6.0 (15.0%)	6.0 (14.0%)	0.892
Type 2 diabetes	2.0 (5.0%)	3.0 (7.0%)	0.341
Heart failure	0 (0.0%)	1.0 (2.3%)	0.167
Sudden cardiac arrest	2.0 (5.0%)	1.0 (2.3%)	0.514
Atrial fibrillation	15.0 (37.5%)	13.0 (30.2%)	0.480
Pregnancy	3.0 (7.5%)	0	0.067
Right-sided AP	34.0 (85.0%)	25.0 (58.1%)	0.007
Left-sided AP	4.0 (10.0%)	16.0 (37.2%)	0.004
Complex right and left-sided AP	2.0 (5.0%)	2.0 (4.7%)	0.941
Right posteroseptal AP	17.0 (42.5%)	12.0 (27.9%)	0.164
Para-Hisian AP	9.0 (22.5%)	6.0 (14.0%)	0.312
Multiple AP	3.0 (7.5%)	3.0 (7.0%)	0.927
Wide AP	2.0 (5.0%)	4.0 (9.3%)	0.449
Permanent pre-excitation	32.0 (80.0%)	30.0 (69.8%)	0.284
Intermittent pre-excitation	3.0 (7.5%)	7.0 (16.3%)	0.200
Concealed AP	5.0 (12.5%)	6.0 (14.0%)	0.845

**Table 2:** Procedural data and results (the values are means ± standard deviation). DAP—dose area product, EAM—electroanatomic system, RF—radiofrequency.

Procedural Data	EAM Group (*n* = 40)	Control Group (*n* = 43)	*p*
Procedure duration (minutes)	93.0 ± 58.3	127.6 ± 57.5	0.009
Application duration (minutes)	13.4 ± 14.8	14.5 ± 14.7	0.884
Number of applications	12.8 ± 14.8	14.6 ± 14.7	0.174
Fluoroscopy duration (minutes)	0	11.6 ± 9.7	<0.001
DAP dose (cGy × cm^2^)	0	1518.5 ±1346.3	<0.001
CARTO	28 (70.0%)	30 (69.8%)	0.982
Ensite	12 (30.0%)	13 (30.2%)	0.982
RF ablation	31 (77.5%)	36 (83.7%)	0.473
Cryoablation	8 (20.0%)	6 (14.0%)	0.462
RF ablation + cryoablation	1 (2.5%)	1 (2.3%)	0.959
First-time ablation	25 (62.5%)	27 (62.8%)	0.978
Acute procedural success	38 (95.0%)	39 (90.7%)	0.449
Long-term success	36 (90.0%)	36 (83.7%)	0.399
Major complications	0	0	0
Minor complications	1 (2.5%)	0	0.138

## Data Availability

Raw data are available upon request to the corresponding author.
